# Erste Ergebnisse der Evaluation eines digitalen Rollators

**DOI:** 10.1007/s00391-022-02118-3

**Published:** 2022-10-07

**Authors:** Ina Dupret, Johannes Gräske, Rebecca Venn, Dagmar Renaud

**Affiliations:** 1 Department Gesundheit und Pflege, Sozialwissenschaftliche Fakultät, Hochschule für Technik und Wirtschaft des Saarlandes, Goebenstraße 40, 66117 Saarbrücken, Deutschland; 2 Fachbereich 2 Gesundheit und Erziehung, Alice Salomon Hochschule Berlin, Berlin, Deutschland

**Keywords:** Elektronikbox am herkömmlichen Rollator, Assistenzsysteme, Sturzangst, Mobilität, Soziale Teilhabe, Electronic box on the conventional rollator, Assistive technology, Fear of falling, Mobility, Social participation

## Abstract

**Hintergrund:**

Zur Kompensation physiologischer Einschränkungen und zum Erhalt der Mobilität im Alter werden bevorzugt Rollatoren genutzt. Allerdings können Hindernisse der Umgebung oder die eingeschränkte Wahrnehmung von Sturzgefahren zu Sturzereignissen führen.

**Ziel der Arbeit:**

Im Rahmen einer Studie wird eine Elektronikbox evaluiert, mit deren Hilfe herkömmliche Rollatoren mit verschiedenen technischen Assistenzsystemen (z. B. Hilferuf an Kontaktpersonen, Sturzerkennung) ergänzt werden (DigiRoll). Die Elektronikbox wurde in der Pilotphase der Studie mit dem Ziel der Weiterentwicklung auf ihre Alltagstauglichkeit getestet.

**Methodik:**

In einem qualitativen Design wurde der DigiRoll in einer Pilotphase mit 10 Teilnehmenden getestet. Die Personen wurden sowohl in Fokusgruppen als auch Einzelinterviews befragt. Zusätzlich erfolgten ethnografische Beobachtungen. Die Auswertung erfolgte qualitativ inhaltsanalytisch.

**Ergebnisse:**

Die Proband:innen zeigten ein großes Interesse an der Entwicklung der Assistenzsysteme zur Steigerung des Sicherheitsgefühls, zugleich wurde die Notwendigkeit der Anpassung des DigiRoll an individuelle Bedarfe deutlich, in Abhängigkeit von der Wohnsituation, der Art der Beeinträchtigung und den persönlichen Lebensgewohnheiten.

**Diskussion:**

Der DigiRoll hat das Potenzial, Menschen mit eingeschränkter Mobilität nachhaltig zu unterstützen, indem er das Sicherheitsgefühl der Nutzer:innen erhöht. Weitere Kontextfaktoren wie der Zugang zu geeigneten Rollatoren und sicheren Gehwegen können nicht durch den DigiRoll beeinflusst werden.

## Abstract (en)

**Background:**

Rollators are preferred to compensate physiological limitations and to maintain mobility in old age; however, environmental obstacles or limited perception of fall hazards can lead to fall events.

**Objective:**

The present study evaluates an electronic box that is used to supplement conventional rollators with various technical assistance systems (e.g. call for help to contact persons, fall detection). The rollator equiped with the electronic box (DigiRoll) was tested for its suitability for everyday use during the pilot phase of the study, with the aim of further development.

**Methods:**

In a qualitative design, the DigiRoll was tested in a pilot phase with 10 participants. Individuals were interviewed in focus groups and individual interviews. In addition, ethnographic observations were made. The data evaluation was carried out using qualitative content analysis.

**Results:**

The test persons showed great interest in the development of the assistance systems to increase the feeling of safety. At the same time, the necessity of adapting the DigiRoll to individual needs became clear, depending on the living situation, the type of impairment and personal habits.

**Conclusion:**

The DigiRoll has the potential to provide sustainable support for people with reduced mobility by increasing the users’ sense of safety. Other contextual factors such as access to suitable rollators and safe walkways cannot be influenced by the DigiRoll.

**Sturzereignisse können für alte Menschen schwerwiegende Verletzungen zur Folge haben, teils verbunden mit Funktionsverlusten, Pflegebedürftigkeit oder sogar Mortalität **[1, 13]**. Neben den physischen sind auch die psychosozialen Auswirkungen von großer Bedeutung. Häufig resultiert Sturzangst, die zu weiterer Einschränkung der Mobilität und körperlichen Aktivität führt und das Sturzrisiko somit erhöht **[20]**. In der Folge reduzieren viele Betroffene damit einhergehend ihre sozialen Aktivitäten **[17]**.**

Etwa die Hälfte (51,6 %) der 70- bis 85-Jährigen gibt Einschränkungen der funktionalen Gesundheit an [4]; häufig ist hiervon die Mobilität betroffen. Beeinträchtigungen der Mobilität können dazu führen, dass Aktivitäten aufgegeben werden und die gesellschaftliche Teilhabe eingeschränkt wird [16]. Hilfsmittel zur Kompensation altersphysiologischer Einschränkungen nehmen zum Erhalt der Mobilität eine wichtige Rolle ein. Als wesentlicher Bestandteil der Sturzprophylaxe vermitteln Rollatoren Menschen mit Mobilitätseinschränkungen ein Gefühl der Sicherheit und fördern die Mobilität [1]. Mit Rollatoren werden auch Sturzrisiken assoziiert: Die Nutzer:innen können an Hindernissen in der Umgebung hängen bleiben, dabei das Gleichgewicht verlieren und stolpern bzw. fallen [10]. Durch eine nachlassende Sehkraft im Alter wird das Sturzrisiko verstärkt [1], insbesondere bei schlechten Lichtverhältnissen.

Digitale Assistenzsysteme stellen einen Ansatzpunkt zur Förderung der Selbstständigkeit älterer Menschen dar. Zur Unterstützung von Menschen mit einer Beeinträchtigung der Mobilität wurden in internationalen Forschungsprojekten bereits digitale Rollatoren entwickelt [2, 19]. Diesen Modellen ist gemeinsam, dass sie auf einer Interaktion zwischen Mensch und Roboter-Rollator als künstliche Intelligenz basieren.

Im Rahmen der vorliegenden Studie wurde eine Elektronikbox entwickelt und unter Alltagsbedingungen getestet, die herkömmliche Rollatoren mit geringem technischem Aufwand und niedrigen Kosten mit Signal- und Alarmtechniken ausstattet. Die Elektronikbox, die verschiedene Assistenzsysteme enthält, wird bei der Zielgruppe am bereits vorhandenen Rollator befestigt (Abb. 1 und 2). Der mit der Elektronikbox ausgestattete Rollator (DigiRoll) soll die Nutzer:innen in ihrem Alltag unterstützen, Sicherheit vermitteln und somit zur Mobilität sowie zur sozialen Teilhabe beitragen. Im Gegensatz zu den oben genannten Modellen stützt sich der DigiRoll nicht auf künstliche Intelligenz. Zur Kostenminimierung wurden kommerzielle Sensoren zur Abstandsmessung sowie einfache Vibrations‑, Lage- und Helligkeitssensoren verwendet.

**Figure 1:**
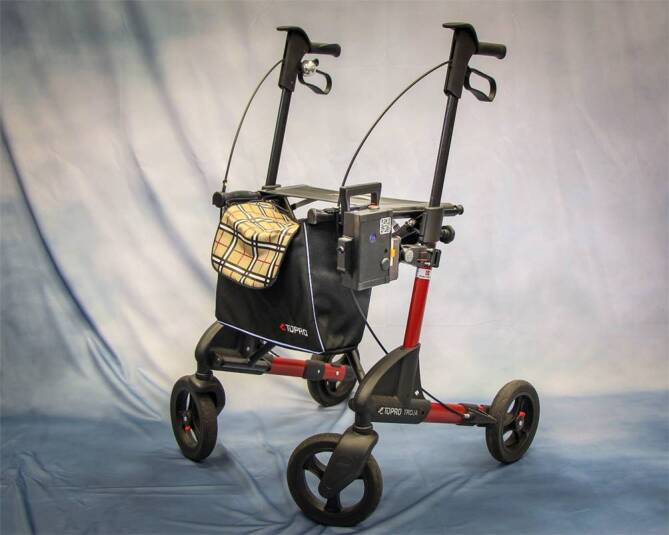

**Figure 2:**
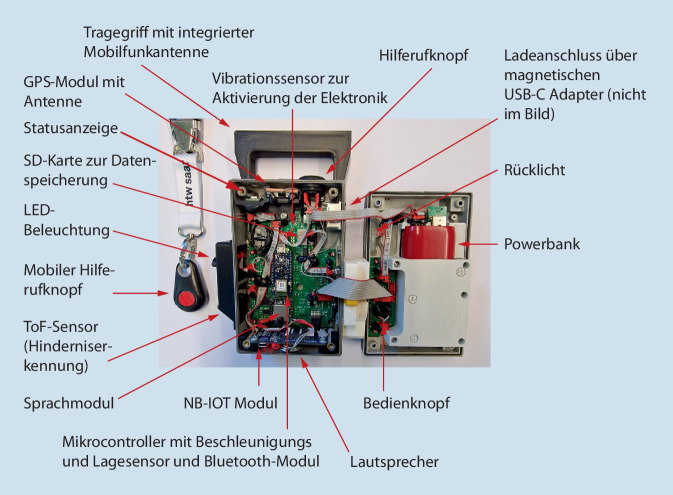

Eine bei Dämmerung automatisch angehende Gehwegbeleuchtung und ein Rücklicht verbessern die Sicht und Sichtbarkeit im Verkehr. Ein Modul zur optischen Abstandsmessung kann Hindernisse und Stufen erkennen und soll durch die Weitergabe eines akustischen und optischen Signals das Sturzrisiko verringern. Bei einem Sturz des Rollators setzt der DigiRoll einen automatischen Hilferuf an festgelegte Personen ab. Ein manueller Hilferuf ist ebenfalls möglich. Im Falle eines Hilferufes werden die Global Positioning System-Daten (GPS-Daten) der Elektronikbox mitgesendet. Wenn die Nutzer:innen vermisst werden, können autorisierte Kontaktpersonen die Position abfragen.

Die Elektronikbox schaltet sich in Abhängigkeit von der Bewegung des Rollators selbstständig ein und aus. Ein in die Elektronikbox integrierter Lautsprecher mit Sprachausgabe weist die Nutzer:innen auf bestimmte Ereignisse hin (z. B. „Sturz erkannt“). Eine Statusanzeige informiert über die verschiedenen Betriebsarten (z. B. Ruhemodus oder Hilferuf). Je nach Intensität der Nutzung muss der Akku der Elektronikbox alle 3 bis 7 Tage geladen werden.

Vor Durchführung einer randomisierten kontrollierten Studie wurde der DigiRoll in einer Pilotphase getestet. Ziele der Pilotphase waren die Überprüfung der Praxistauglichkeit der Assistenzsysteme unter Alltagsbedingungen sowie die technische Optimierung anhand der Bedarfe der Nutzer:innen.

## Methodik

### Design

Um die subjektiven Erfahrungen im Umgang mit Rollatoren allgemein sowie spezifisch nach Testung der Elektronikbox zu erheben, wurde für die Pilotphase ein qualitatives Design gewählt.

### Sample

Auf Basis eines „purposive sampling“ [14] wurden insgesamt 10 Personen rekrutiert, die einen eigenen Rollator im Alltag regelmäßig nutzten. Eine erste Gruppe von 7 Personen testete im Sommer 2020 4 Wochen lang die Elektronikbox unter Alltagsbedingungen am eigenen Rollator (Testphase 1). Die Ergebnisse führten zu einer Modifikation der Elektronikbox. Zum Alltagstest dieser Innovationen wurde eine weitere 2‑wöchige Testphase konzipiert: Im Winter 2020/2021 wurde eine neue Generation der Elektronikbox durch 3 weitere Nutzer:innen getestet (Testphase 2).

### Ablauf des Tests

Nach einer detaillierten Aufklärung über das Projekt wurden die Freiwilligen mit der Elektronikbox ausgestattet. Sie bekamen die Anweisung, ihren Rollator, wie gewohnt, in den Alltag zu integrieren und wurden ausführlich in die Handhabung des DigiRoll eingewiesen. Den Testpersonen wurde eine Bedienungsanleitung ausgehändigt, und es bestand die Möglichkeit, sich bei Rückfragen telefonisch an die Projektmitarbeitenden zu wenden.

### Datenerhebung

Zu Beginn von Testphase 1, vor Montage der Elektronikbox, wurde eine Fokusgruppe [5] durchgeführt, um Daten zur Nutzung des Rollators im Alltag sowie zu den Bedarfen hinsichtlich eines Assistenzsystems zu erfassen. Der inhaltlich vorstrukturierte Leitfaden enthielt Kategorien zur Einbindung des Rollators in den Alltag, zu Barrieren, zur Erfahrung mit Sturz- und Notfallsituationen sowie zur Zufriedenheit mit dem eigenen Rollator (Tab. 1). Nach Beendigung der Testphase 1 wurden die Proband:innen in Einzelinterviews zu ihren Erfahrungen mit dem DigiRoll befragt; der Leitfaden beinhaltete u. a. Fragen zu den Erfahrungen mit der Elektronikbox, insbesondere zur Nützlichkeit und Praxistauglichkeit der einzelnen Assistenzfunktionen. Die Teilnehmenden der Testphase 2 wurden coronabedingt ausschließlich in Einzelinterviews nach Ende der Testphase befragt. Ergänzt wurden die Interviewdaten durch ethnografische Beobachtungen [18]. Im Rahmen der Feldkontakte wurden alle mündlichen Rückmeldungen der Nutzer:innen und relevanten Beobachtungen systematisch in Feldnotiz-Dateien notiert. Voraussetzung für die Teilnahme war die informierte Zustimmung gemäß den datenschutzrechtlichen Bestimmungen; Einverständniserklärungen erfolgten schriftlich.

**Table 1:** 

**Kategorie 1: Nutzung des herkömmlichen Rollators im Alltag**	
*Unterkategorie 1.1: *Mobilität und Teilhabe durch den Rollator	„Alles mache ich mit dem Rollator, und das ist für mich Freiheit“ (ID01, Z91, Testphase 1, t0-Befragung)
*Unterkategorie 1.2: *Barrieren im Wohnumfeld	„Weil ich nur einmal am Tag die Stufen da gehe, mir reicht das, wenn ich so 50 Stufen da hinter mich gebracht habe.“ (ID07, Z249–252, Testphase 1, t1-Befragung)
*Unterkategorie 1.3: *Barrieren im öffentlichen Verkehrsraum	„Diese Blätter liegen da. Da ist eine pappige Substanz, da rutschen Sie.“ (ID03, Z1515, Testphase 1, t0-Befragung)
*Unterkategorie 1.4: *Erfahrungen mit Sturz- und Notfallsituationen	„Ich gehe nirgendwo weit alleine hin, weil ich ja Angst doch um mein Leben ein bisschen habe.“ (ID06, Z285–286, Testphase 1, t1-Befragung)
**Kategorie 2: Praxistauglichkeit des DigiRoll**	
*Unterkategorie 2.1: *Notwendigkeit des Entfernens und Transports der Elektronikbox	„Wenn ich einkaufen gehe, zurückkomme vom Einkaufen, dann muss ich jedes Mal das Gerät hochtragen. Erste Etage. Ich habe die Hände voll und einmal runter und wieder hoch, das ist auch für mich zu anstrengend.“ (ID08, Z5–6, Testphase 1, t1-Befragung)
*Unterkategorie 2.2: *Wendigkeit des DigiRoll	„Man muss aufpassen, wenn man so um die Kurve oder die Ecken fährt. Dass man da nix mitnimmt. Denn das Gerät ragt ja so ein bisschen vor.“ (ID07, Z539–540, Testphase 1, t1-Befragung)
*Unterkategorie 2.3: *Bedienung der Elektronikbox	„Ich habe immer gedacht, was will der mir sagen?“ (ID03, Z655, Testphase 1, t1-Befragung)
*Unterkategorie 2.4: *Image des DigiRoll	„Was ist denn das für ein modernes Ding? So haben die Leute reagiert.“ (ID07, Z993–994, Testphase 1, t1-Befragung)
**Kategorie 3: Erfahrungen mit den einzelnen Assistenzsystemen des DigiRoll**	
*Unterkategorie 3.1*: Gehwegbeleuchtung und Rücklicht	„Das ist eine große Hilfe, wenn ich abends spät bei Dunkelheit hier um den Häuserblock gehe. (…) Ja, nicht nur, dass man besser sieht, sondern man wird auch gesehen. Das ist der große Vorteil.“ (ID11, Z330–339, Testphase 2, t1-Befragung)
*Unterkategorie 3.2: *Manueller Hilferuf, automatische Sturzerkennung und GPS-Ortung	„Dass ich mich kann melden, wenn irgendwas ist. Das finde ich gut.“ (ID01, Z300, Testphase 1, t1-Befragung)
*Unterkategorie 3.3*: Hinderniswarnung	„Der piepst immer, wenn er will.“ (ID09, Z277, Testphase 2, t1-Befragung)

### Datenauswertung

Die Interviews wurden digital aufgezeichnet, transkribiert und qualitativ-inhaltsanalytisch ausgewertet [11]. Inhaltstragende Textstellen und Beobachtungen wurden den Kategorien zugeordnet und inhaltlich zusammenfassend dargestellt. Weitere Kategorien wurden induktiv aus dem Material gebildet. Die Analyse erfolgte durch die Autorinnen. Die Ergebnisse wurden verglichen, es zeigten sich weitgehende Übereinstimmungen, Abweichungen wurden diskutiert und im Konsens kodiert. Im Anschluss wurden alle Feldnotizen in das Kategoriensystem eingeordnet. Die Ergebnisse der Interviewauswertung wurden auf diese Weise validiert und empirisch angereichert.

## Ergebnisse

Es nahmen 3 Männer und 7 Frauen teil, die zum Befragungszeitpunkt zwischen 60 und 91 Jahre alt waren und selbstständig in Privathaushalten lebten.

### Nutzung des herkömmlichen Rollators im Alltag

#### Mobilität und Teilhabe durch den Rollator

In den Interviews wurde die herausragende Bedeutung des Rollators für die Bewältigung des Alltags betont. Der Rollator bedeute Freiheit, da er Mobilität und eine Teilhabe am gesellschaftlichen Leben ermögliche: „Alles mache ich mit dem Rollator, und das ist für mich Freiheit“ (ID01, Z91). Überwiegend kam der Rollator im Außenbereich zum Einsatz; 3 der Befragten nutzten ihn zusätzlich in ihrer Wohnung.

#### Barrieren im Wohnumfeld

Alle Testpersonen berichteten von physischen Barrieren im Wohnbereich wie auch im öffentlichen Raum und beschrieben ihre Strategien im Umgang mit diesen Hürden (z. B. Einsatz anderer Hilfsmittel wie einen Gehstock, Meiden des Ortes). Sieben von 10 Befragten hatten keinen barrierefreien Zugang zu ihrer Wohnung und mussten im Alltag Treppenstufen überwinden. Die meisten dieser Personen stellten ihren Rollator im Hausflur ab und nutzten das Treppengeländer als Stütze.

#### Barrieren im öffentlichen Verkehrsraum

Darüber hinaus wurde von den Testpersonen das Problem der Sturzrisiken auf öffentlichen Gehwegen angesprochen: Lose Platten, Unebenheiten im Straßenbelag, fehlende Absenkungen der Bordsteine sowie rutschige Oberflächen (z. B. durch Laub) würden ein sicheres Gehen mit dem Rollator erschweren. Daher müssten sie häufig auf die Straße ausweichen, wo sie sich jedoch einem erhöhten Unfallrisiko aussetzten. Des Weiteren wurde bemängelt, dass schlechte Sichtverhältnisse und kurze Ampelphasen für Rollatorfahrer:innen erhebliche Risiken mit sich brächten. Entsprechend wurde in der Fokusgruppe ein starkes Interesse an der automatischen Gehwegbeleuchtung und an einem Rücklicht zum Ausdruck gebracht. Hiermit wurde die Hoffnung auf mehr Sicherheit im Verkehr verknüpft. In Bezug auf das Assistenzsystem der Hinderniswarnung wurde der Wunsch geäußert, vor kleineren Stolperfallen, wie z. B. vor Unebenheiten im Straßenbelag, gewarnt zu werden. Die vorgesehene Hinderniswarnung war allerdings auf das Erkennen größerer Hindernisse, wie etwa Stufen oder Bordsteine ausgerichtet und entsprach somit nur begrenzt den Bedarfen der Zielgruppe.

#### Erfahrung mit Sturz- und Notfallsituationen

Die Befragten der Fokusgruppe schilderten ihre Angst, bei Stürzen oder in anderen Notfallsituationen auf sich allein gestellt zu sein. Im Zusammenhang mit dem Sturzrisiko wurde häufig auf die Qualität des Rollators und auf große Unterschiede in den Modellen hingewiesen. Zur Absicherung bei Notfällen im häuslichen Umfeld benannten die Befragten Hausnotrufsysteme, welche einige Befragte zum Zeitpunkt der Befragung bereits nutzten. Allerdings wurde kritisch angemerkt, die Reichweite der Funksender decke nicht den Mobilitätsradius der Nutzer:innen ab. Daher bestand ein starkes Interesse an den für Notfälle vorgesehenen Assistenzsystemen des DigiRoll (manueller Hilferuf, Sturzerkennung mit automatischem Hilferuf, GPS-Ortung durch autorisierte Notfallkontakte).

### Praxistauglichkeit des DigiRoll

Die Rückmeldungen der Teilnehmenden der Testphase 1 führten zu zahlreichen Anpassungen der Elektronikbox, sodass – wie im Folgenden dargelegt – in der Testphase 2 die Praxistauglichkeit des DigiRoll gesteigert wurde.

#### Bedienung der Elektronikbox

Die Teilnehmenden der Testphase 1 gaben vielseitige Hinweise zur Optimierung der Bedienung der Elektronikbox: Aufleuchtende Statusanzeigen, Sprachausgaben sowie die Gehwegbeleuchtung sollten z. B. in störenden Momenten manuell abgeschaltet werden können. Diese Anforderung wurde in Testphase 2 durch einen lautlosen Standby-Knopf gelöst. Um Nutzer:innen mit Beeinträchtigung der Hörfähigkeit gerecht zu werden, wurde die Sprachausgabe in Testphase 2 so gestaltet, dass Hörgeräte durch eine vorgeschaltete Ansage („DigiRoll meldet: …“) aktiviert wurden, bevor die Übermittlung der eigentliche Sprachnachricht (z. B. „… Hilferuf erkannt.“) erfolgte. Um den Teilnehmenden den Akkustand anzuzeigen, wurde in der Testphase 2 die Ladeanzeige der integrierten Powerbank visualisiert. In der Pilotphase 2 fiel die Bewertung der Statusanzeigen, der Sprachausgaben und der Powerbank positiv aus.

#### Notwendigkeit des Entfernens und Transports der Elektronikbox

Alle Testpersonen, die ihren Rollator im Hausflur stehen ließen, entfernten die Elektronikbox regelmäßig vom Rollator, um diese mit in die Wohnung zu nehmen. Der Transport der Elektronikbox, zusätzlich zu den Einkäufen und anderen Gegenständen, wurde von den Teilnehmenden der Testphase 1 als Belastung empfunden; zudem fiel einigen das Entfernen und Befestigen der Elektronikbox schwer. Daher wurde die Elektronikbox in der Pilotphase 2, zur Zufriedenheit der Testpersonen, mit einem Tragegriff versehen, das Gewicht reduziert und der Befestigungsmechanismus vereinfacht.

#### Wendigkeit des DigiRoll

Einige Testpersonen bemängelten eine eingeschränkte Wendigkeit des Rollators durch die Elektronikbox. So seien sie u. a. beim Einkaufen an Regalen hängen geblieben, „weil das Gerät so weit raus ragt“ (ID07, Z508). Zur Zufriedenheit der Teilnehmenden wurde in Testphase 2 die Größe der Elektronikbox angepasst und die Halterung näher an der Stange des Rollators positioniert.

#### Image des DigiRoll

Insgesamt zeigten sich die Proband:innen gegenüber den technischen Potenzialen des DigiRoll aufgeschlossen. Einige der Beteiligten schilderten eine ideelle Aufwertung des Rollators: Demzufolge sei die Nutzung eines Rollators an Stigmatisierungserfahrungen gekoppelt; dank der Elektronikbox würden sie sich „modern fühlen“ (ID03, Z463). Zugleich äußerten einzelne Teilnehmende die Furcht vor einer Überwachung durch den digitalen Rollator. Vor diesem Hintergrund wurde bei der Entwicklung der Elektronikbox auf Kamera- und Spracherkennungsmodule verzichtet. In einem ausführlichen Aufklärungsgespräch wurde versichert, dass der DigiRoll nicht in der Lage ist, Stimmen oder Bilder aufzuzeichnen. Bezüglich der GPS-Ortung wurde klargestellt, dass kein Bewegungsprofil erstellt wird und die GPS-Position nicht öfter als 4‑mal täglich durch autorisierte Kontaktpersonen abgefragt werden kann.

### Erfahrungen mit den einzelnen Assistenzsystemen

#### Gehwegbeleuchtung und Rücklicht

Die automatische Gehwegbeleuchtung und das Rücklicht wurden von den Nutzer:innen äußerst positiv gewertet: Die Rollatorfahrer:innen begrüßten die verbesserte Sicht und die erhöhte Sichtbarkeit. Es wurden einzelne Justierungen bezüglich der Intensität und Ausrichtung des Lichtkegels vorgeschlagen.

#### Manueller Hilferuf, automatische Sturzerkennung und GPS-Ortung

In Bezug auf den manuellen Hilferuf, die automatische Sturzerkennung und die GPS-Ortung wurde von den Testpersonen angemerkt, dass diese Assistenzsysteme zu einer erheblichen Steigerung des Sicherheitsempfindens beitragen könnten. Auf Wunsch der Proband:innen wurde in der Testphase 2 der Hilferufknopf an der Elektronikbox durch einen tragbaren Hilferufknopf ergänzt, für den Fall, dass der Rollator bei einem Sturzereignis wegrollt, der Sturz demnach nicht erkannt wird und der manuelle Hilferufknopf außer Reichweite ist. Trotz dieser Modifikationen sahen die Proband:innen zum Ende der Testphase 2 noch Entwicklungsbedarf, so bestanden Zweifel an der Zuverlässigkeit der Übermittlung der Hilferufe an die Notfallkontakte. Dabei wurde die Tatsache bemängelt, dass von den Notfallkontakten nicht erwartet werden könne, immer unmittelbar ihre SMS oder E‑Mails einzusehen, sodass ein Hilferuf leicht übersehen werden könne. Weiterhin wurde die Sorge geäußert, dass die automatische Sturzerkennung zu Fehlalarmen führt. Daher wurden eine Sprechfunktion mit den Kontaktpersonen sowie eine Koppelung des Hilferufes an Dienstleister:innen (Hausnotruf, Leitstelle) vorgeschlagen.

#### Hinderniswarnung

Während der Testphase 2 wurde die Elektronikbox zusätzlich mit einer Hinderniswarnung ausgestattet. Hier zeigte sich die Herausforderung, die akustischen und optischen Warnsignale so einzustellen, dass diese von den Nutzer:innen rechtzeitig wahrgenommen werden, ohne aber im Alltag als störend empfunden zu werden. Die optimale Einstellung hing von der sensorischen Empfindlichkeit jedes Einzelnen ab und davon, ob die Nutzer:innen die Hinderniswarnung überhaupt zum Erkennen von Stolperfallen benötigten. Für die Feldphase wurde dementsprechend entschieden, dass die Hinderniswarnung optional ein- oder ausgeschaltet werden kann.

## Diskussion

Die Pilotstudie diente vor Durchführung der Feldphase einem ersten Test auf Praxistauglichkeit unter Alltagsbedingungen sowie einer bedarfsorientierten, technischen Optimierung. Aufgrund der Rückmeldungen der Testpersonen wurden kontinuierlich Anpassungen vorgenommen und eine neue Generation des DigiRoll entwickelt. Dabei stellte sich heraus, dass dieser Prozess zum Ende der Pilotphase noch nicht abgeschlossen war. In Analogie zu anderen Forschungsprojekten zu teilhabefördernden digitalen Assistenzsystemen manifestierte sich die Notwendigkeit einer Konzeption der Technologieentwicklung als „iterativen Gestaltungsprozess“ [8].

Die Ergebnisse der Pilotphase zeigen, dass auf die individuellen Bedürfnisse zugeschnittene, sichere Rollatoren für die Zielgruppe einen zentralen Faktor bei der Bewältigung des Alltags darstellen. Die Nutzer:innen erleben den Rollator grundsätzlich als Mittel zum Gewinn von Freiheit. Die Teilnehmenden äußerten zudem ein starkes Interesse an der Entwicklung digitaler Assistenzsysteme, mit denen u. a. das Sturzrisiko und die Sturzangst verringert sowie die Sicherheit im Straßenverkehr erhöht werden könnten. Besonders positiv wurden im Praxistest des DigiRoll die automatische Gehwegbeleuchtung und das Rücklicht bewertet, welche das Sicherheitsgefühl durch verbesserte Sicht und Sichtbarkeit im Straßenverkehr erhöhten. Dagegen fand das Assistenzsystem der Hinderniswarnung bei den Testpersonen wenig Anklang oder wurde sogar als störend empfunden. Konsequenterweise wurde für die Feldphase eine Vorrichtung zur Abschaltung der Hinderniswarnung integriert. Zu einem stärkeren Sicherheitsgefühl trugen die manuelle und automatische Hilferuffunktion sowie die GPS-Ortung bei, obgleich die Proband:innen zum Ende der Pilotphase diesbezüglich noch Weiterentwicklungsbedarf sahen. Auch wenn z. B. eine gewünschte Anbindung an professionelle Dienste im Rahmen des Projektes nicht realisierbar war, liegt der Vorteil des Systems bereits in dieser Generation der Elektronikbox darin, dass mit geringem finanziellen Aufwand ein Hilferuf an autorisierte Kontaktpersonen gesendet werden kann.

Die Ergebnisse der Pilotphase verdeutlichen, dass eine Anpassung des DigiRoll an individuelle Bedarfe nötig ist, um dessen Praxistauglichkeit zu garantieren. Auch in anderen Forschungskontexten zu teilhabefördernden Technologien wurde dieses Phänomen hervorgehoben: Die Heterogenität der Zielgruppen, kombiniert mit einer großen Diversität der Anwendungskontexte, führt demzufolge zur Notwendigkeit einer Anpassung der Technologien an individuelle Bedarfe (vgl. 8). Bei der individuellen Konfiguration des DigiRoll müssten Faktoren wie z. B. die Wohnsituation (Barrierefreiheit) und persönliche Lebensgewohnheiten (z. B. Bus- oder Autofahrten) berücksichtigt werden. Lautstärke bzw. Lichtintensität der optischen und akustischen Signale sollten auf individuelle Beeinträchtigungen (eingeschränkte Hör- oder Sehfähigkeit) eingestellt werden können. Gleichwohl besteht das Dilemma, den Nutzer:innen einerseits eine kontextabhängige, individuelle Steuerung der Assistenzsysteme zu garantieren und zugleich die Funktionsweise der Elektronikbox möglichst einfach und verständlich zu konzipieren.

Eine Herausforderung bei der Entwicklung teilhabefördernder Technologien liegt in der Unterschätzung der Komplexität des Technikeinsatzes in der Versorgung [8, 9]. Voraussetzung für eine nachhaltige Nutzung digitaler Assistenzsysteme ist deren „Einbettung in Versorgungspraktiken und -strukturen“ [12]. Es müssen weitere Akteure in die Versorgungsstrukturen einbezogen und dabei auch der Transfer von Wissen über die Technologien garantiert werden [7]. Vor diesem Hintergrund erscheint einerseits vielversprechend, dass zum Ende der Pilotphase des Projektes DigiRoll eine Technologie bereitstand, die von den Nutzer:innen weitgehend selbstständig gehandhabt werden konnte. Andererseits muss eingeräumt werden, dass eine Weiterentwicklung zur Marktfähigkeit die Bereitstellung eines nachhaltigen Systems zur Unterstützung der Nutzer:innen bei der Handhabung des DigiRoll erfordert.

Die Ergebnisse der Befragungen illustrieren darüber hinaus, dass das Sturzrisiko und die subjektive Sicherheit von Rollatorfahrer:innen von externen Kontextfaktoren abhängen, die durch den DigiRoll nicht beeinflusst werden können. Hierzu zählen der Zugang zu geeigneten Rollatoren, aber auch Unfallrisiken im innerstädtischen Verkehrsraum, wie Sturzrisiken auf Gehwegen durch lose Platten, Laub etc. Auch zu kurze Ampelphasen oder Sturzrisiken bei Busfahrten sind wichtige Kontextfaktoren, die mit den im Projekt entwickelten Assistenzsystemen nicht verändert werden können. Diese Kontextfaktoren haben einen beträchtlichen Einfluss auf die potenzielle Wirkungsentfaltung des DigiRoll und verweisen auf die Grenzen von Strategien, die bei einer individuellen Kompensation von Beeinträchtigungen durch technische Assistenzsysteme ansetzen. Daraus kann die Empfehlung abgeleitet werden, Ansätze zur Entwicklung assistiver Technologien, mit Strategien zum Abbau von Barrieren in der Umwelt zu kombinieren [6].

Überrascht hat der Befund, dass die Elektronikbox als Imageaufwertung des Rollators erlebt wurde und somit zu einer Überwindung einer gefühlten Stigmatisierung als Rollatorfahrer:in beiträgt. Zugleich muss aber auch die Angst vor Überwachung durch die Elektronikbox bedacht werden. Angesichts der teils vorherrschenden Befürchtung, durch die Elektronikbox abgehört zu werden, wurde auf eine Umsetzung des Wunsches einiger Testpersonen, die Elektronikbox mit einer Gegensprechfunktion auszustatten, verzichtet. Diese Entscheidung verweist auf die Notwendigkeit einer Abwägung zwischen den konfligierenden Bedürfnissen nach Sicherheit und der Wahrung der Privatsphäre der Betroffenen [3, 15].

### Limitationen

Bei der Weiterentwicklung des DigiRoll müssen ethische und rechtliche Aspekte der Nutzung kommerzieller Sensoren weitervertieft werden. Das betrifft insbesondere die GPS-Ortung, deren Zuverlässigkeit bei der Ortung sowie beim Datenschutz nicht durchgehend garantiert werden kann. Eine weitere Limitation stellt die Tatsache dar, dass der DigiRoll coronabedingt während der Pilotphase noch nicht in stationären Einrichtungen getestet werden konnte, was nun in der Feldphase erfolgt. Angesichts des abweichenden Kontextes ist zu erwarten, dass sich hier neue Forschungsfragen und technische Optimierungsbedarfe ergeben. Insbesondere müssen pflegeorganisatorische Aspekte bei der Anbindung an interne Kommunikationssysteme sowie bei der technischen Begleitung der Zielgruppe beachtet werden.

## Fazit für die Praxis

- Rollatoren haben aus der Sicht der Nutzer:innen eine herausragende Bedeutung bei der Bewältigung des Alltags.
- Durch das Assistenzsystem der Gehwegbeleuchtung und des Rücklichts konnte das Sicherheitsgefühl der Rollatorfahrer:innen gesteigert werden.
- Vonseiten der Zielgruppe bestand ein großes Interesse an der Entwicklung eines Hilferufsystems mit hoher Reichweite
- Bei der Entwicklung der Hilferufsysteme muss zwischen dem Bedürfniss der Nutzer:innen nach Sicherheit und dem Wunsch nach einer Wahrung der Privatsphäre abgewogen werden.
- Bei einer Weiterentwicklung der Elektronikbox sollte eine individuelle Konfiguration der Elektronikbox ermöglicht werden, da teils heterogene Bedarfe bezüglich der Ausgestaltung der Assistenzsysteme vorliegen.
